# Relationship between the EQ-5D index and measures of clinical outcomes in selected studies of cardiovascular interventions

**DOI:** 10.1186/1477-7525-7-96

**Published:** 2009-11-26

**Authors:** Kimberley A Goldsmith, Matthew T Dyer, Peter M Schofield, Martin J Buxton, Linda D Sharples

**Affiliations:** 1 Papworth Hospital NHS Trust, Cambridge, UK; 2 MRC Biostatistics Unit, Institute of Public Health, Cambridge, UK; 3 Institute of Psychiatry, King's College London, UK; 4 Health Economics Research Group, Brunel University, Uxbridge, UK; 5 National Collaborating Centre for Mental Health, The Royal College of Psychiatrists, UK

## Abstract

**Background:**

The EuroQoL 5D (EQ-5D) has been widely used in studies of cardiac disease, but its measurement properties in this group are not well established. The study aimed to quantify the relationship between measures commonly used in studies of cardiac disease and the EQ-5D index across different levels of disease severity.

**Methods:**

Patient-level data from 7 studies of cardiac interventions were used, which included randomised trials and observational studies. Relationships between the EQ-5D index and commonly used cardiac measures, Canadian Cardiovascular Society (CCS) angina severity class, treadmill exercise time (ETT) and scales of the Seattle Angina Questionnaire (SAQ) were examined. Mixed effects linear regression was used to assess these relationships, with the EQ-5D index as the response.

**Results:**

Study sample sizes ranged from 68 to 2419. Mean baseline EQ-5D index ranged from 0.77 in patients at diagnosis (95% CI 0.75, 0.78) to 0.43 in patients with advanced disease (95% CI 0.39, 0.48) and differed significantly across studies (p < 0.001). There was evidence of a ceiling effect in patients at diagnosis. The minimum clinically important difference of a one minute increase in ETT was associated with a 0.019 (95% CI 0.014, 0.025) increase in EQ-5D index. One class increase in CCS was associated with a 0.11 (95% CI 0.09, 0.13) decrease in EQ-5D index. A 10 unit increase in SAQ scales was associated with increases between 0.04 and 0.07 in EQ-5D index (95% CIs 0.03, 0.05 and 0.05, 0.08). Tests of heterogeneity indicated the EQ-5D-covariate relationships were consistent across levels of disease severity for ETT and the treatment satisfaction scale of the SAQ, but heterogeneous for age, gender, CCS angina class and other scales of the SAQ.

**Conclusion:**

The EQ-5D index varies with coronary disease severity. The relationship between the EQ-5D index and an outcome measure used in cardiac intervention studies, ETT, was consistent across disease severity levels, but the relationship between demographic variables, CCS angina class and most of the SAQ scales and the EQ-5D index was heterogeneous for patients with different levels of coronary disease. Differences in the EQ-5D index associated with clinically important differences in cardiac measures can be quantified and vary between three important examples - angina class, ETT and SAQ.

## Background

**Figure 1:**
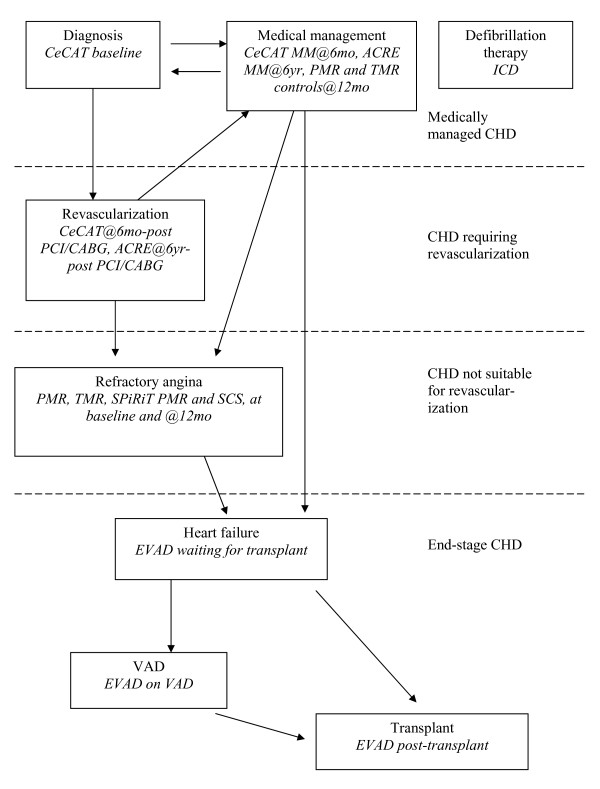
**Coronary heart disease (CHD) schematic**. Key: MM - medical management, PCI - balloon angioplasty ± stenting, CABG - bypass surgery.

Coronary heart disease (CHD) is common and new treatments for patients in various stages of the disease continue to be developed and evaluated. Figure 1 shows a schematic of how patients may move between different levels of severity of CHD. Patients diagnosed with CHD can either be managed medically (which can maintain a similar level of disease to when they were diagnosed), with a cardiological procedure such as balloon angioplasty/stenting (PCI), or with surgical revascularization (coronary artery bypass grafting - CABG) [1]. Following revascularization, the vast majority of patients have a good symptomatic response, and those patients generally return to being medically managed. Other patients may not be suitable for revascularization at the time of diagnosis and will progress to refractory angina [2]. A different group of patients suffering from electrophysiological problems of the heart may have a defibrillator inserted. Many of the patients in these different groups could be susceptible to eventual heart failure, which in selected patients could lead to heart transplantation (Tx) with or without the use of a ventricular assist device (VAD) to support heart function in the interim [3]. As new interventions for cardiac patients with different levels of disease severity are developed, they are often tested in clinical trials against current treatment options.

Clinical trial-based evaluations of treatments in many fields, including cardiology, often include cost-effectiveness, which requires the elicitation of health related quality of life (HRQoL) from patients in order to calculate quality-adjusted life years (QALYs). The EuroQoL 5D (EQ-5D) is a questionnaire that provides a generic measure of HRQoL [4-6]. Responses from the questionnaire can be converted to a single health index utility score [7] and can be used in conjunction with survival data to calculate QALYs. The index ranges from -0.59 to 1 in the UK [8], where the value for death is 0 and negative index values represent health states valued worse than death. The EQ-5D index is widely known and used, and is currently recommended by the National Institute for Health and Clinical Excellence as a tool for measuring adult patients' perception of utility [6,9].

The EQ-5D index has often been used to assess HRQoL and to calculate QALYs for cost-effectiveness analyses in trials of interventions in cardiac patients [3,10-12] and has been found to be valid and reliable in these patients [13-20]. Ceiling effects of the EQ-5D index where good health states are poorly discriminated have, however, been seen in cardiac patients [20]. A recent analysis of the literature has shown that EQ-5D index scores are variable in examples of patients with cardiovascular disease (Dyer M, Goldsmith, K, Sharples, L, Buxton, M: A review of health utilities using the EQ-5D in studies within the cardiovascular area, submitted). The review showed that mean EQ-5D index scores ranged from 0.45 to 0.88, and 0.31 to 0.78 in studies of ischaemic heart disease (IHD) and heart failure patients, respectively. The review also showed that many individual studies have looked at the responsiveness of EQ-5D index to treatment and found that scores generally increase with improvements after treatment as measured by Canadian Cardiovascular Society (CCS) angina severity class or New York Heart Association (NYHA) classification (Dyer M, Goldsmith, K, Sharples, L, Buxton, M: A review of health utilities using the EQ-5D in studies within the cardiovascular area, submitted). Preliminary meta-regression of aggregate data from these studies showed a large amount of heterogeneity in EQ-5D index scores after stratifying for angina class, which was not explained by different types of disease (Dyer M, Goldsmith, K, Sharples, L, Buxton, M: A review of health utilities using the EQ-5D in studies within the cardiovascular area, submitted).

Consistency in relationships between the EQ-5D index, patient characteristics and cardiac outcome measures across different studies/disease severity groups have not been assessed using patient level data. This study aims to use individual patient data to assess how the EQ-5D index varies in cardiac patients with different levels of disease severity and to explore and quantify the relationship between the EQ-5D index and both patient characteristics and outcome measures commonly used in cardiac studies, such as exercise treadmill time (ETT), CCS angina classification and Seattle Angina Questionnaire (SAQ) scales.

## Methods

### The EQ-5D index

The EQ-5D questionnaire consists of 5 questions covering the following health domains: mobility, self-care, usual activity, pain and anxiety/depression [4-6]. Participants are asked to choose their level of problems in each domain from three options: no problems, some or moderate problems and severe problems. The questionnaire also includes a visual analog scale allowing the participant to rate their current health state from 0-100. The 5 health domain questions can be used to generate a single index value or utility by applying societal preference weights to states of health as elicited by the questionnaire [4-7]. These preference weights and an algorithm for calculating the EQ-5D index were determined in a UK population using data from the Measurement and Valuation of Health survey [7].

### Choice of studies

In order to be able to study effects at the patient level, the data used were limited to those from studies that the investigators had been involved in, so that the relationship between the EQ-5D index and cardiac outcome measures could be examined using patient level records. This was, therefore, an opportunistic sample that was not obtained through a systematic review. All studies were conducted in the UK and the UK scoring algorithm for the EQ-5D index was used.

Studies were further chosen to be able to study patients across the spectrum of disease by including those that had collected EQ-5D data from cardiac patients with different severities of CHD. The relationship between the EQ-5D index and measures of cardiac outcomes was the primary focus, so it was also important that the studies used measured the cardiac outcomes of interest, including ETT, CCS angina class and the SAQ, which are further described below. Some studies collected NYHA rather than CCS. The relationship between the EQ-5D index and the Short Form 6D (SF-6D), another utility measure used in cost-effectiveness analysis [21], was also studied. This latter relationship was not of direct interest as it has been studied previously for patients with other types of diseases [22] and the focus was on the relationship between patient characteristics and the EQ-5D index, not that between different measures of HRQoL. The aim in studying the SF-6D was both to compare our results to previous findings, and to quantify the relationship in cardiac patients for completeness.

The study includes secondary analysis of results from a range of clinical trials. All primary clinical trials had ethical approval from Local Research Ethics committees between 1993 and 2001.

### Cardiac outcome measures

The ETT is a validated clinical test used to assess suspected or known CHD. The test follows the Bruce protocol which requires walking on a treadmill at a given speed and with a given grade, both of which increase through three stages [23]. The modified protocol uses a constant lower speed and lower grades (all 1.7 mph with: Stage 1 - 0% grade; Stage 2 - 5% grade; Stage 3, which is equivalent to Stage 1 in the regular Bruce protocol - 10% grade), and is often used in patients that are elderly, sedentary, or have known heart disease.

The CCS is a rating scale for stable angina [24]. It ranges from 0, meaning no symptoms, to Class IV for the worse symptoms [See Additional File 1]. The NYHA is a more general cardiac disease rating scale, which is similar to CCS, but not completely consistent with it [See Additional File 1] [25].

The SAQ consists of 11 questions that can be converted into 5 scales assessing functional status for patients with angina: exertional capacity (ECS), anginal stability (ASS), anginal frequency (AFS), disease perception (DPS) and treatment satisfaction (TSS) [26]. The SAQ has been validated and widely used in studies of patients with CHD [26,27].

### Studies used for the analysis

Seven studies of cardiac interventions conducted in the UK were used. The studies are summarized in Figure 1 and Table 1. Patients ranged from those undergoing imaging for suspected coronary disease (diagnosis stage) to those with severe disease. Using studies in different types of patients allowed us to examine relationships at different stages of disease (Figure 1 and Table 1). We were also able to study effects in patients having different treatments by dividing observations into different disease/treatment groups using data gathered within the studies at different time intervals (Table 1). Age and gender were recorded for all studies at study entry. The studies included:

**Table 1:** Summary of studies used and disease/treatment group and treatment variables used in regression models

Name	Short form	Inclusion/Exclusion Criteria	Study type	Study size	Cardiac subgroup	Disease/ treatment groups (random effect)	Treatment
Cost-effectiveness of functional cardiac testing in the diagnosis and management of CHD [12]	CECaT	I: established or suspected CHD referred for angiography E: recent MI, revascularization, urgent need for revascularization, contraindications to study tests	Diagnosis/ management (RCT)	898	Coronary disease diagnosis	CECaT baseline CECaT MM CECaT PCI CECaT CABG	Pre-treatment MM PCI CABG

Appropriateness for coronary revascularization [1]	ACRE	I: Consecutive patients having coronary angiography E: None	Diagnosis/ management (cohort)	2419	Coronary revascularization	ACRE MM ACRE PCI ACRE CABG	MM PCI CABG

Implantable Cardioverter Defibrillator (ICD) therapy in different patient groups [28]	ICD	I: patients implanted with an ICD at Papworth or Liverpool hospitals between 1991 and 1999 and a random sample of those implanted in 2000 and 2001	Diagnosis/ management (cohort)	229	Cardiac arrythmias	ICD	ICD

Percutaneous myocardial revascularization (PMR) compared to continued medical therapy [29]	PMR	I: angina refractory to medication or revascularization E: implanted devices, significant comorbidity, contraindications to study treatments	Angina (RCT)	73	Angina	PMR	Pre-treatment* MM PMR

Transmyocardial laser revascularization (TMR) compared to continued medical therapy [30]	TMR	I: angina refractory to medication or revascularization E: implanted devices, significant comorbidity, contraindications to study treatments	Angina (RCT)	188	Angina	TMR baseline TMR MM TMR	Pre-treatment* MM TMR

Spinal cord stimulation (SCS) compared to PMR [31]	SPiRiT	I: angina refractory to medication or revascularization E: implanted devices, significant comorbidity, contraindications to study treatments	Angina (RCT)	68	Angina	SPiRiT baseline PMR SCS	Pre-treatment* PMR SCS

Evaluation of ventricular assist devices (VAD) patients compared to patients on transplant waiting list (Tx WL) [3]	Tx WL	I: a sample of patients listed for transplant between April 2002 and December 2004	Heart failure (cohort)	47	Heart failure	Tx WL	Pre-treatment*
	VAD	I: all patients with VADs implanted as part of NSCAG funded program between April 2002 and December 2004	Heart failure (cohort)	35	Heart failure	VAD Post-tx (post-transplant)	VAD Tx

Key: CHD - coronary heart disease, I - inclusion criteria, E - exclusion criteria, MI - myocardial infarction, RCT - randomised controlled trial, MM - medical management, PCI - balloon angioplasty/stenting, CABG - coronary artery bypass graft, NSCAG - National Specialist Commissioning Advisory Group

*NB: Pre-treatment for that study, but these patients will not be treatment naïve.

Cost-effectiveness of functional cardiac testing in the diagnosis and management of CHD (CECaT) [12]: a randomised controlled trial (RCT) of coronary disease diagnostic methods in patients presenting for angiography. The EQ-5D index, ETT, CCS, SAQ and SF-6D were measured at randomisation, 6 months post-treatment and 18 months post-randomisation. Diagnostic methods were randomised, not treatments; treatments were given as part of routine patient management. The treatment options were medical management (MM), PCI or CABG. The first treatment a patient had was used to classify them into one of these three treatment groups. Measurements made at study entry were classed as pre-treatment and the 6 month post-treatment measurements were taken as treatment measurements in the three treatment groups.

Appropriateness for coronary revascularization (ACRE) [1]: a prospective cohort study in patients presenting for angiography. The EQ-5D index was measured only at the 6 year follow-up point. CCS and SF-6D were measured at study entry and the 6 year follow-up point. The full SAQ was administered at study entry, while only the questions for calculating the ASS and AFS scales of the SAQ were asked at the 6 year follow-up point. Patients were treated as indicated clinically with MM, PCI, or CABG. As we were only using data from the 6 year time point due to the availability of the EQ-5D index, the ACRE study only contributed post-treatment patients (although baseline information has been summarized). Patients could have had multiple different types of treatment over the 6 year follow-up so patients were classed according to the invasiveness of the treatment as follows: if a patient had CABG any time over the course of the study, they were in the CABG group, if the patient had only had PCI but not CABG, they were in the PCI group, and if the patient had neither, they were in the MM group.

Implantable Cardioverter Defibrillator (ICD) therapy in different patient groups (ICD) [28]: a cross-sectional study in a cohort of patients implanted with an ICD at one of two centres between 1991 and the end of 2001. Sixty-nine percent of the patients that had an ICD implant - all of those still alive who were implanted between 1991 and 1999 and a random sample of those still alive who were implanted in 2000 and 2001 - were sent the EQ-5D questionnaire, with a 73% response rate (229 patients). Because patients had been implanted over a span of time, the EQ-5D measurement was made at variable times post-implant. This measurement was considered to be a treatment measurement for ICD and pre-treatment measurements were not available. NYHA was collected from patient notes, just before or at implant.

Percutaneous myocardial revascularization compared to continued medical therapy (PMR) [29]: a RCT of PMR for refractory angina not relieved by medical management. Patients were randomised to receive PMR or MM and were followed up at 3, 6 and 12 months. The EQ-5D index, ETT, CCS, SAQ and SF-6D were measured at all follow-up points. Measurements made at study entry were classed as pre-treatment. Measurements made 12 months post-surgery in the PMR group, and post-assessment in the MM group, were taken as treatment measurements for PMR and MM.

Transmyocardial laser revascularization compared to continued medical therapy (TMR) [30]: a RCT of TMR for refractory angina not relieved by medical management. Patients were randomised to receive TMR or MM and were followed up at 3, 6 and 12 months. The EQ-5D index, ETT, CCS and SF-6D were measured at all follow-up points. Measurements made at study entry were classed as pre-treatment. Measurements made 12 months post-surgery in the TMR group, and post-assessment in the MM group, were taken as treatment measurements for TMR and MM.

Spinal cord stimulation (SCS) compared to PMR (SPiRiT) [31]: an RCT of PMR versus SCS for refractory angina not relieved by medical management. Patients were randomised to receive PMR or SCS and were followed up at 3, 12 and 24 months. The EQ-5D index, ETT, CCS, SAQ and SF-6D were measured at all follow-up points. Measurements made at study entry were classed as pre-treatment. Measurements made 12 months post-treatment in the PMR and SCS groups were taken as treatment measurements for these two groups.

Evaluation of ventricular assist devices (VAD) patients compared to patients on transplant waiting list (Tx WL) (EVAD) [3]: an observational cohort study - evaluation of VADs in heart failure patients and a comparison group of patients on the Tx WL. In this case, measurements taken in the waiting list group pre-transplantation were classed as pre-treatment. Measurements taken in the VAD group pre-transplantation were taken as treatment measurements for the VAD group. Post-transplantation measurements in both groups in the subset of patients that underwent transplantation were taken as treatment measurements for transplantation (Tx). Measurements of EQ-5D, NYHA and SF-6D were taken at several time points, so the earliest one after acceptance on to the transplant list, implant with a VAD, or Tx, was used.

### Statistical analysis

The EQ-5D index and other continuous variables were summarized using the mean and standard deviation and boxplots. Categorical variables were summarized using frequencies and proportions. The difference in baseline EQ-5D index across studies was examined using a general linear model with the EQ-5D index as the outcome and study as the predictor using only data gathered pre-treatment (at study entry).

General linear mixed models were used to assess the relationship between the EQ-5D index and a series of explanatory variables, allowing for heterogeneity across the disease/treatment groups, which are described above and in Table 1. In each model EQ-5D_*ij *_for patient *j *(*j *= 1, ..., n_*i*_) in disease/treatment group *i *(*i *= 1,..., 20) was used. Not all 20 groups had all explanatory variables, so *i *varied depending on the number of groups who had the given variable available. The explanatory variables of primary interest were age, sex, ETT, CCS and the scales of the SAQ. SF-6D was also studied. A separate analysis was undertaken for each explanatory variable. Age, ETT, the scales of the SAQ and the SF-6D were centred at their mean value (for age, mean age at baseline) in the models. For all explanatory variables, a fixed effect and a Normal random effect was assumed. In addition, the treatment applied (pre-treatment, MM, PCI, CABG, ICD, PMR, TMR, SCS, VAD, Tx) and the study type (Diagnosis/management, Angina, Heart failure) were included as fixed effects (Table 1). Thus an example of the models would be:

Where:

*α*_0 _is a fixed intercept,

*α*_1_, *α*_2 _and *α*_3 _are fixed effects coefficients

*β*_*i*_~*N*(0, *σ*_*β*_^2^) are random effects allowing for different age effects in different disease/treatment groups, and

*ε*_*ij*_~*N*(0, *σ*_*ε*_^2^) represents residual random error not explained by the other terms in the model.

After models were fit, the importance of the treatment and study type fixed effects were tested by removing each variable from each model in turn and using a conditional F-test [32] to compare models with and without these covariates.

The minimally important difference (MID) in the EQ-5D index has been estimated to be between 0.05 - 0.07 [33,34], and was assumed to be 0.05 in the primary analyses of many of the studies used here. Changes in ETT and CCS that have been considered clinically important differences in many of the cardiac studies described above were a one minute change in ETT and a two class change in CCS class. For SAQ, a 10 unit change is considered clinically significant [26]. In this study we assessed the change in EQ-5D index for a ten year increase in age, males versus females, a one minute increase in ETT, a one class increase in CCS, a 10 unit increase in the SAQ scales and a 0.1 unit change in SF-6D as these seemed reasonable quantities across which to quantify differences in the EQ-5D index. NYHA data gathered in the ICD and EVAD studies were not included in modelling because only two studies gathered this data.

Cochran's Q test statistic [35] and the I^2 ^statistic [36] were used to assess heterogeneity between disease/treatment groups. In a meta-analysis context, the Cochran's Q allows for a statistical test of heterogeneity between studies by taking the sum of the squared differences of each study from the pooled estimate, weighted in the same way in which studies were weighted to get the pooled estimate. I^2 ^uses Cochran's Q statistic and the degrees of freedom of the test to provide a measure of the percent of total variation that is due to heterogeneity between studies, or here, between disease/treatment groups.

## Results

Study sample sizes ranged between 68 and 2419 (Table 1). The EQ-5D index had more of a ceiling effect in healthier patients being diagnosed with heart disease (CECaT trial) as opposed to those that were symptomatic [See Additional File 2]. Study subjects were mostly male (69% or greater, Table 2) and in studies of heart failure the patients were younger on average than patients in the other studies (Table 2). Patients being diagnosed with heart disease had higher EQ-5D index scores, ECS, AFS, DPS and SF-6D scores and longer exercise times than patients with more advanced disease at study enrolment [See Additional Files 2 and 3]. Mean baseline EQ-5D index was higher in patients at earlier stages of disease progression, such as those in the CECaT trial (mean EQ-5D index 0.77), than they were in the patients with later-stage disease in the other trials (the lowest values were for patients with angina, for example, 0.43 in the TMR trial, Table 2). The EQ-5D index differed significantly between these pre-treatment groups (p < 0.001). The EQ-5D index score was generally higher post-treatment, with more pronounced ceiling effects [See Additional File 2]. SF-6D increased slightly and ETT was about the same post-treatment [See Additional File 2]. Most of the scores on the SAQ scales also increased post-treatment [See Additional File 3].

**Table 2:** Patient characteristics at baseline by study

Characteristic	CECaT n = 898	ACRE n = 2419	PMR n = 73	TMR n = 188	SPiRiT n = 68	EVAD Tx WL n = 47
Mean baseline EQ-5D (SD)	0.77 (0.22)	---	0.48 (0.30)	0.43 (0.29)	0.44 (0.30)	0.51 (0.27)

Mean age (SD)	62 (9.4)	60 (9.7)	62 (6.4)	60 (7.6)	64 (8.4)	48 (11.7)

Gender						

Male (%)	619 (69)	1701 (70)	69 (95)	169 (90)	60 (88)	39 (83)

Female (%)	279 (31)	718 (30)	4 (5)	19 (10)	8 (12)	8 (17)

Diabetes						

Yes (%)	36 (4)	263 (11)	N/A	33 (18)	6 (9)	N/A

No (%)	862 (96)	2156 (89)	N/A	155 (82)	62 (91)	N/A

Previous heart attack/angioplasty/ revascularization						

Yes (%)	342 (38)	N/A	71 (97)	185 (98)	67 (99)	N/A

No (%)	556 (62)	N/A	2 (3)	3 (2)	1 (1)	N/A

CCS or NYHA class*						

0 (%)	59 (7)	258 (11)	---	---	---	

I (%)	191 (21)	185 (8)	---	---	---	---

II (%)	536 (60)	496 (21)	---	---	---	---

III (%)	100 (11)	211 (9)	48 (66)	143 (76)	47 (69)	18 (38)

IV (%)	12 (1)	639 (26)	25 (34)	43 (23)	21 (31)	7 (15)

Key: CECaT - Cost-effectiveness of functional cardiac testing in the diagnosis and management of coronary heart disease study, ACRE - Appropriateness for coronary revascularization study, PMR - Percutaneous myocardial revascularization compared to continued medical therapy study, TMR - Transmyocardial laser revascularization compared to continued medical therapy study, SPiRiT - Spinal cord stimulation (SCS) compared to PMR study, EVAD - Evaluation of ventricular assist devices (VAD) patients compared to patients on transplant waiting list (Tx WL) study, EQ-5D - Euroqol 5D, SD - standard deviation, CCS - Canadian Cardiovascular Society angina classification, NYHA - New York Heart Association angina classification

*CCS class for all but EVAD groups. In the case where percentages do not sum to 100, it is due to missing values.

**Figure 2:**
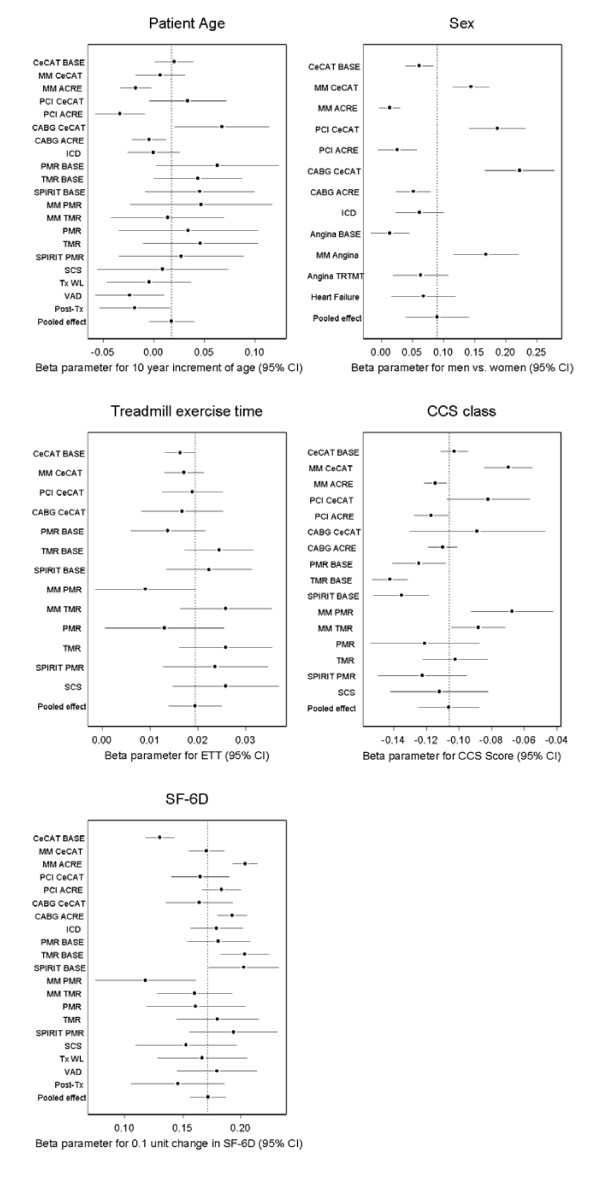
**Relationship between the EQ-5D index and patient characteristics/clinical outcome measures across diagnosis groups**. Key: CECaT - Cost-effectiveness of functional cardiac testing in the diagnosis and management of coronary heart disease study, BASE - baseline measurements, MM - medical management, ACRE - Appropriateness for coronary revascularization study, PCI - percutaneous angioplasty/stenting, CABG - coronary artery bypass graft, ICD - Implantable Cardioverter Defibrillator, PMR - Percutaneous myocardial revascularization compared to continued medical therapy study, TMR - Transmyocardial laser revascularization compared to continued medical therapy study, SPiRiT - Spinal cord stimulation (SCS) compared to PMR study, Tx WL - transplant waiting list, VAD - ventricular assist device, Tx - post heart transplantation, Angina = data from PMR, TMR and SPiRiT studies, TRTMT = data from all treatments in Angina studies, Heart failure = TxWL and VAD patients, CCS - Canadian Cardiovascular Society angina classification, SF-6D - short form 6D.

**Figure 3:**
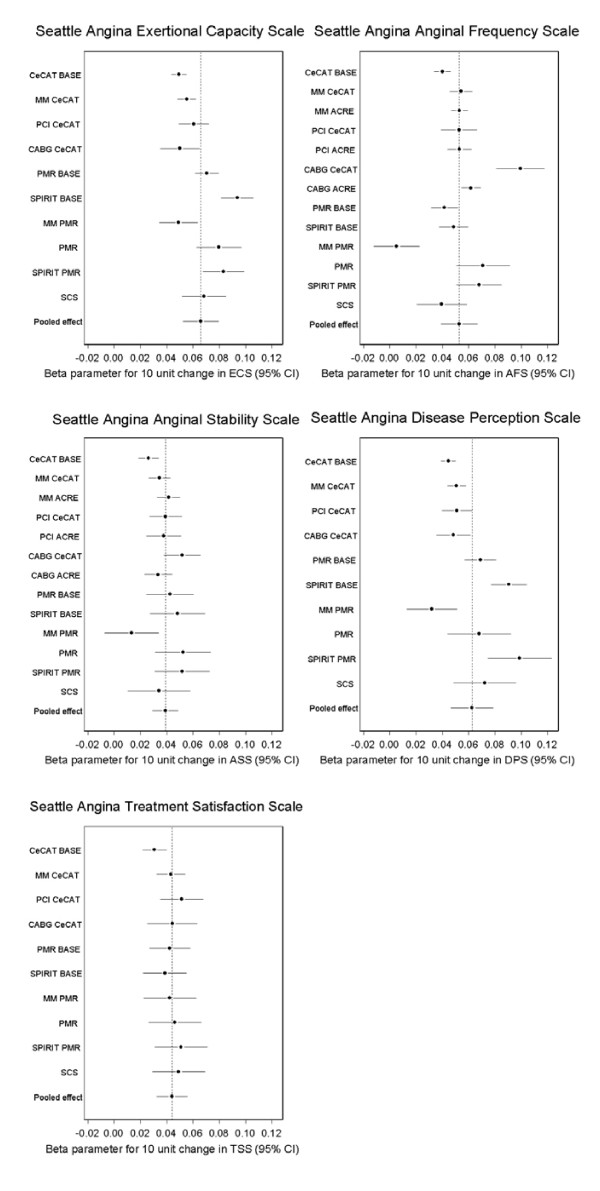
**Relationship between the EQ-5D index and Seattle Angina Questionnaire scales across diagnosis groups**. Key: CECaT - Cost-effectiveness of functional cardiac testing in the diagnosis and management of coronary heart disease study, BASE - baseline measurements, MM - medical management, PCI - percutaneous angioplasty/stenting, CABG - coronary artery bypass graft, PMR - Percutaneous myocardial revascularization compared to continued medical therapy study, SPiRiT - Spinal cord stimulation (SCS) compared to PMR study, ECS - exertional capacity scale, ACRE - Appropriateness for coronary revascularization study, ASS - angina severity scale, AFS - anginal frequency scale, TSS - treatment satisfaction scale, DPS - disease perception scale.

Overall there was a small positive non-significant relationship between age and EQ-5D index with older patients having higher EQ-5D index scores (Table 3 and Figure 2 - the forest plots in Figures 2 and 3 show the β parameter and 95% CI for the given variable for each disease/treatment group and the pooled effect of the given variable across the groups). There was, however, significant heterogeneity (I^2 ^= 61%) between studies (Table 3). In the two cohort studies (ACRE and EVAD) there was a negative relationship whereby EQ-5D index scores decreased with age, while in the four RCTs (CECaT, TMR, PMR, Spirit) EQ-5D index scores increased with age.

**Table 3:** Relationship between variables and the EQ-5D index - pooled effect and heterogeneity from evidence synthesis across studies

Variable	Pooled effect (95% CI)	Heterogeneity as measured by I^2^, p-value
Age (10 year increment)	0.02 (-0.01, 0.04)	61%, < 0.001

Sex (Men vs. women)	0.09 (0.04, 0.14)	93%, <0.001

ETT (1 minute increment)	0.019 (0.014, 0.025)	36%, 0.10

CCS (1 class increase)	0.11 (0.09, 0.13)	86%, <0.001

SAQ - ECS (10 unit increment)	0.066 (0.053, 0.079)	87%, <0.001

SAQ - ASS (10 unit increment)	0.039 (0.029, 0.049)	51%, 0.02

SAQ - AFS (10 unit increment)	0.052 (0.039, 0.067)	87%, <0.001

SAQ - TSS (10 unit increment)	0.044 (0.032, 0.056)	0, 0.45

SAQ - DPS (10 unit increment)	0.063 (0.047, 0.079)	87%, <0.001

SF-6D (0.10 unit increment)	0.17 (0.16, 0.19)	83%, <0.001

Key: EQ-5D - EuroQol 5D, I^2 ^- I^2 ^index for quantifying heterogeneity, ETT - Treadmill exercise test, CCS - Canadian Cardiovascular Society angina classification, SAQ - Seattle Angina Questionnaire, ECS - exertional capacity scale, ASS - angina severity scale, AFS - anginal frequency scale, TSS - treatment satisfaction scale, DPS - disease perception scale, SF-6D - short form 6D

In the case of gender, male patients had better EQ-5D index scores than women (0.09 units greater in men on average, Table 3), but the magnitude of the relationship was not consistent across disease/treatment groups (Table 3 and Figure 2).

ETT had a small positive relationship with the EQ-5D index, where the EQ-5D index increased by 0.019 (95% CI 0.014, 0.025) for each minute increase in ETT (Table 3 and Figure 2). The relationship between ETT and the EQ-5D index did not exhibit a large amount of heterogeneity across groups (I^2 ^= 36%).

CCS class had a large negative relationship with the EQ-5D index, with a decrease of 0.11 (95% CI 0.09, 0.13) with each CCS class increase (Table 3 and Figure 2), and this relationship exhibited a large amount of heterogeneity across disease/treatment groups (Table 3). In general, there was a stronger relationship between CCS class and EQ-5D index in angina trials pre-treatment than in the other disease/treatment groups.

For the SAQ, the EQ-5D index increased by between approximately 0.04 and 0.07 for a 10 unit increase in the different SAQ scales (Table 3 and Figure 3). The proportion of variation due to disease/treatment heterogeneity was high and significant for the scales that measured ability to exert oneself, anginal frequency and perception of disease (ECS, AFS and DPS, I^2 ^all equal to 87%), but was lower for angina severity (ASS) (Table 3). There was no heterogeneity observed in the relationship between angina treatment satisfaction (TSS) and the EQ-5D index (Table 3).

As expected, there was large positive relationship between the EQ-5D index and the other generic measure of HRQoL studied, SF-6D. A large proportion of the variation in the EQ-5D/SF-6D relationship was due to heterogeneity across disease/treatment groups (Table 3 and Figure 2).

Study and treatment type fixed effects were important covariates for almost all of the patient variables of interest (data not shown), and so were left in all models for consistency.

## Discussion

This project utilized data from several different studies of cardiovascular patients to assess the relationship between the EQ-5D index and various patient characteristics and outcomes. Using studies from a range of clinical scenarios allowed us to assess relationships between the EQ-5D index and other variables at different cardiac disease stages and in different treatment groups. A patient-level analysis such as this has substantially more power to detect effects than a meta-regression of aggregate results, and allows effects to be measured with greater precision.

We observed ceiling effects of the EQ-5D index, especially in cardiac patients in the diagnosis stage of disease, and also after treatment. Ceiling effects in the EQ-5D index have been shown in cardiac patients and for other groups [22,37,38]. Healthier patients, such as those from the CECaT study, also exhibited weaker associations between predictor variables and the EQ-5D index in many cases and the effects differed in general in patients studied as a cohort (ACRE, ICD, EVAD - patients with heart failure and transplant recipients) from those in patients selected for RCTs. Patients included in RCTs are highly selected. There is some evidence for worse risk profiles [39] and higher mortality [39,40] in non-participants versus participants in cardiac trials. Cohorts, on the other hand, tend to be less exclusive. It could be that patients selected for randomised trials are healthier and are a more homogeneous group than those included in cohort studies. This could lead to less variability in the variables of interest in these patients, and in particular, the ceiling effect may constrain the variation in EQ-5D index scores. In the patients studied here, the EQ-5D index scores for the CECaT trial groups were less variable than those in the comparable ACRE groups. It has also been noted that there are gaps in utility scores near the upper limit of 1 for the EQ-5D index [38], suggesting that the EQ-5D index does not discriminate well between good health states. The ceiling effect and decreased sensitivity of the EQ-5D index at the upper end of the range will need to be kept in mind when studying patients early in the course of cardiac disease and patients post-treatment.

### EQ-5D index and age

The relationship between the EQ-5D index and age varied between patient groups, although the pooled effect was small and not statistically significant. In patients recruited to trials, the EQ-5D index increased with age, which is contrary to the effect seen in "normal" populations [37,41]. In cohort studies (ACRE, EVAD, ICD), the EQ-5D index decreased with increasing age, as expected. Beyond the general differences between trial and cohort patients described above, older patients selected for RCTs may have better than average quality of life for their age/sex group, ie trial patients in older age groups may be particularly heavily selected and would usually exclude those with co-morbidities. Cohort patients, such as those with heart failure, were less selected so that they were more like people in the general population with respect to the relationship between age and the EQ-5D index.

### EQ-5D index and sex

Men had higher EQ-5D index scores than women. In some population studies, women reported more problems on the EQ-5D [37,41] than men, but this did not lead to significantly lower index scores in the UK population [8]. Women with CHD have also been shown to score lower on the disease-specific HRQoL measure, SAQ [42]. There is evidence that women presenting with stable angina or acute coronary syndromes have higher levels of risk factors than men, including CCS or NYHA class [43,44], suggesting women may be presenting later in the course of disease. This was also shown to be the case in a trial of ICDs [45]. The relationship between sex and EQ-5D index was stronger in CECaT study patients after treatment and angina patients on medical management. Effects were smaller in cohort patients and angina patients pre-treatment and post-treatment. Most of the variation (92.9%) across the disease/treatment groups was due to heterogeneity.

### EQ-5D index and ETT

For each minute increase in ETT, there was a 0.019 increase in EQ-5D index. Based on a recommended MID of 0.05 between health states [33], it seems there is not a strong relationship between ETT and the EQ-5D index. The relationship between these two variables was much more consistent than the relationships with most other variables across disease/treatment groups. The EQ-5D assesses mobility but also a number of other elements contributing to quality of life and so reflects more non-physical aspects of HRQoL, while ETT is an indicator of physical limitations, perhaps explaining the small magnitude of the relationship.

### EQ-5D index and CCS

For CCS angina class, CECaT treatment groups and angina trials groups treated with routine medical management showed a smaller relationship between EQ-5D index and CCS than CECaT and angina groups at study entry and ACRE treatment groups. As in the case of the other relationships explored here, this may be partly due to different levels of heterogeneity in trial and cohort participants. In some studies (the angina trials, for example), most patients were in CCS classes III and IV, meaning less variability in this measure. There was a relatively strong relationship between CCS and EQ-5D index. This could be because CCS is a discrete measure and a one-class change may correspond to a relatively large difference in functional limitations.

### EQ-5D index and SAQ

Increases in the scales of the SAQ, which indicate improvements in different aspects of angina, were associated with increases in EQ-5D index greater than the MID for exertional capacity, anginal frequency and disease perception, while anginal stability and treatment satisfaction were associated with slightly smaller differences of approximately 0.04. Taken together, these indicate a reasonably strong relationship between the generic EQ-5D HRQoL measure and the disease-specific SAQ. For most of the SAQ scales (and some other variables studied), there was a smaller relationship between the scale and EQ-5D index in the PMR MM group. This was a small group with few low EQ-5D index scores, and this lack of variation may explain the different results for this group. The ECS had a smaller relationship with EQ-5D index in the CECaT groups than in most of the angina groups, perhaps reflecting greater physical disability in the angina groups allowing for larger changes in the EQ-5D index. The relationship between anginal frequency and EQ-5D index was at, or greater than, the MID for most groups. The effects were larger in the CECaT CABG group and to some extent in the ACRE CABG groups and the PMR treatment groups, perhaps because patients in these groups were treated by more aggressive means and perceived a large decrease in anginal episodes soon after treatment. For disease perception, there was a larger effect for patients in the angina trials, possibly reflecting the specificity of the scale for angina/concern about having a heart attack, which may be less relevant for healthier CECaT patients, for example. This sort of inconsistency in the relationship between both the CCS and the SAQ and the EQ-5D index across disease/treatment groups could reflect that while some cardiac patients suffer from angina, recently diagnosed patients and heart failure patients are less likely to have pain from angina at rest or with mild activity. Treatment satisfaction was not very variable and had a consistent relationship with the EQ-5D index. The ASS had a similar relationship with EQ-5D index in most groups, and in fact, with the PMR MM group removed, heterogeneity in this measure across disease/treatment groups was lower and only borderline significant (44%, p = 0.053).

### EQ-5D index and SF-6D

The relationship between EQ-5D index and SF-6D in angina patients was explored for completeness, and was strong, as might be expected for two measures used for the same purpose. There was an increase of 0.17 in EQ-5D index for each 0.10 unit increase in SF-6D. This relationship has been explored before for patients in different disease groups using ordinary linear regression and with SF-6D as the outcome [22], and when applying a similar model to our data, we obtained a similar result (data not shown). There was a large amount of heterogeneity in the relationship across disease/treatment groups, which was not necessarily expected given that these are both composite measures of HRQoL. It has been previously noted, however, that there are differences between these two measures [20,22,38].

### Limitations

A limitation of the study is its focus on patients recruited to randomised trials. While this does not affect the internal validity of the results, it may limit their generalisability to the overall population with CHD. Secondly, CCS class was studied in models as a continuous variable, whereas it is a discrete measure. This could be a reason for the large effect size of CCS. This was necessary in part because there were few or no patients in the lower CCS classes in studies in patients with advanced disease. Thirdly, differences by sex were difficult to assess separately for some disease/treatment groups, angina in particular, because there was a small proportion of women in many of the studies, so further work could be done in studies with more women to assess the robustness of the estimate of the relationship between sex and EQ-5D index in cardiac patients. Finally, we were not able to assess the relationship between other measures such as the Health Utilities Index or the Minnesota Living with Heart Failure score and the EQ-5D index as these are not generally used in the UK and were not therefore available in the datasets that were used.

## Conclusion

We have used individual patient level data to show that the EQ-5D index decreases as cardiac disease severity increases and that the EQ-5D index has a ceiling effect in patients with mild CHD and after treatment. The EQ-5D index has a relatively strong relationship across different levels of CHD severity with sex, the scales of the SAQ and CCS angina severity class and a smaller relationship with age and ETT. The variation seen in the relationship between the EQ-5D index and these variables, with the exception of the ETT and treatment satisfaction measured by SAQ, is in large part due to heterogeneity between groups of patients with different levels of CHD severity.

## Competing interests

The authors declare that they have no competing interests.

## Authors' contributions

KG performed the analysis and drafted and edited the manuscript. MD edited the manuscript. PS managed patients in the studies and edited the manuscript. MB designed the study and edited the manuscript. LS designed the study and extensively edited the manuscript. All authors read and approved the final manuscript.

## Supplementary Material

Additional file 1 **Canadian Cardiovascular Society (CCS) angina and New York Heart Association (NYHA) functional capacity and objective assessment of patients with diseases of the heart classification systems**. The table outlines the definitions of the different classification levels of the CCS and NYHA classifications of heart disease. Click here for file

Additional file 2 **Boxplots of the EQ-5D index and other patient characteristics pre-treatment and after treatment by study**. The figure shows boxplots of the raw baseline and post-treatment values of EQ-5D, SF-6D and exercise treadmill time. Click here for file

Additional file 3 **Boxplots of Seattle Angina scale scores pre-treatment and after treatment by study**. The figure shows boxplots of the raw baseline and post-treatment values of the scales of the Seattle Angina Questionnaire. Click here for file
