# Supplementary material for: Relationship between the EQ-5D index and measures of clinical outcomes in selected studies of cardiovascular interventions
Source: Health Qual Life Outcomes. 2009 Nov 26;7:96. doi: 10.1186/1477-7525-7-96 (PMC2789057; doi:10.1186/1477-7525-7-96)
Supplement: Additional file 1 — Canadian Cardiovascular Society (CCS) angina and New York Heart Association (NYHA) functional capacity and objective assessment of patients with diseases of the heart classification systems. The table outlines the definitions of the different classification levels of the CCS and NYHA classifications of heart disease. [file 1477-7525-7-96-S1.DOC]

Additional File 1. Canadian Cardiovascular Society (CCS) angina and New York Heart Association (NYHA) functional capacity and objective assessment of patients with diseases of the heart classification systems

| Classification level | CCS | NYHA |
| --- | --- | --- |
| 0 | No symptoms | --- |
| I | Angina only with strenuous exertion or prolonged activity | Cardiac disease, but no symptoms, no limitation or shortness of breath when walking or climbing stairs |
| II | Angina only during vigorous activity or under stressful circumstances, slight limitation to activities | Slight limitation of physical activity. Mild angina/shortness of breath/fatigue during ordinary activity |
| III | Angina on walking 1 or 2 blocks on the level or climbing one flight of stairs, moderate limitation to activities | Marked limitation of physical activity, angina/shortness of breath/fatigue when walking short distances, comfortable only at rest |
| IV | Any physical activity causes angina, may be angina at rest, severe limitation of activities | Severe limitation, angina/shortness of breath/fatigue at rest, worsens with activity. |
