# Supplementary material for: Relationship between the EQ-5D index and measures of clinical outcomes in selected studies of cardiovascular interventions
Source: Health Qual Life Outcomes. 2009 Nov 26;7:96. doi: 10.1186/1477-7525-7-96 (PMC2789057; doi:10.1186/1477-7525-7-96)
Supplement: Additional file 2 — Boxplots of the EQ-5D index and other patient characteristics pre-treatment and after treatment by study. The figure shows boxplots of the raw baseline and post-treatment values of EQ-5D, SF-6D and exercise treadmill time. [file 1477-7525-7-96-S2.PDF]

Baseline values | Values after treatment

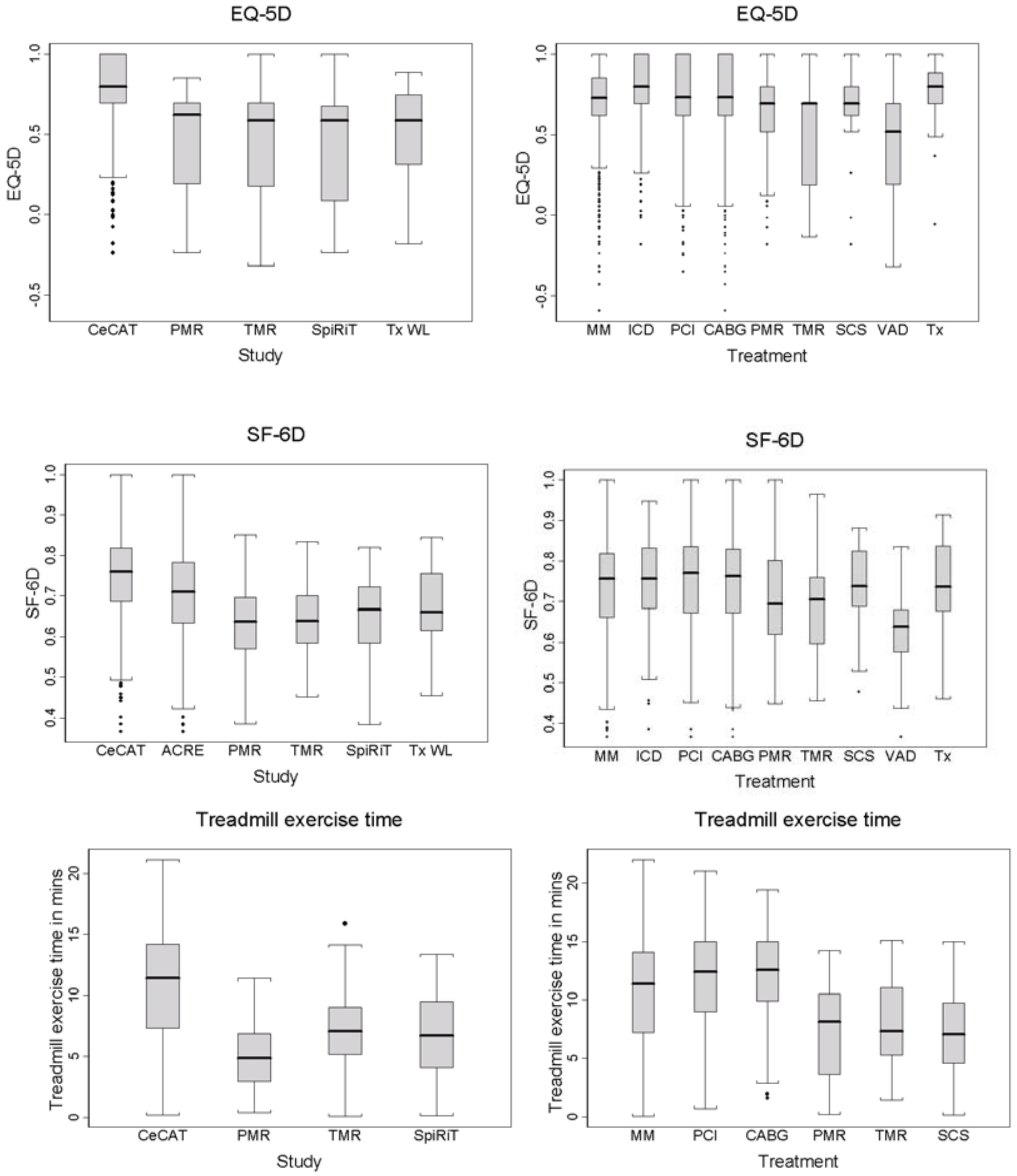

Key: EQ-5D – Euroqol 5D, CeCAT – Cost-effectiveness of functional cardiac testing in the diagnosis and management of coronary heart disease study, PMR – Percutaneous myocardial revascularization compared to continued medical therapy study, TMR – Transmyocardial laser revascularization compared to continued medical therapy study, SpiRiT – Spinal cord stimulation (SCS) compared to PMR study, Tx WL – transplant waiting list, ACRE – Appropriateness for coronary revascularization study, SF-6D – short form 6D, MM – medical management, ICD – Implantable Cardioverter Defibrillator, PCI – percutaneous angioplasty/stenting, CABG – coronary artery bypass graft, VAD – ventricular assist device, Tx – post heart transplantation
