# Supplementary material for: Relationship between the EQ-5D index and measures of clinical outcomes in selected studies of cardiovascular interventions
Source: Health Qual Life Outcomes. 2009 Nov 26;7:96. doi: 10.1186/1477-7525-7-96 (PMC2789057; doi:10.1186/1477-7525-7-96)
Supplement: Additional file 3 — Boxplots of Seattle Angina scale scores pre-treatment and after treatment by study. The figure shows boxplots of the raw baseline and post-treatment values of the scales of the Seattle Angina Questionnaire. [file 1477-7525-7-96-S3.PDF]

Baseline values

Values after treatment

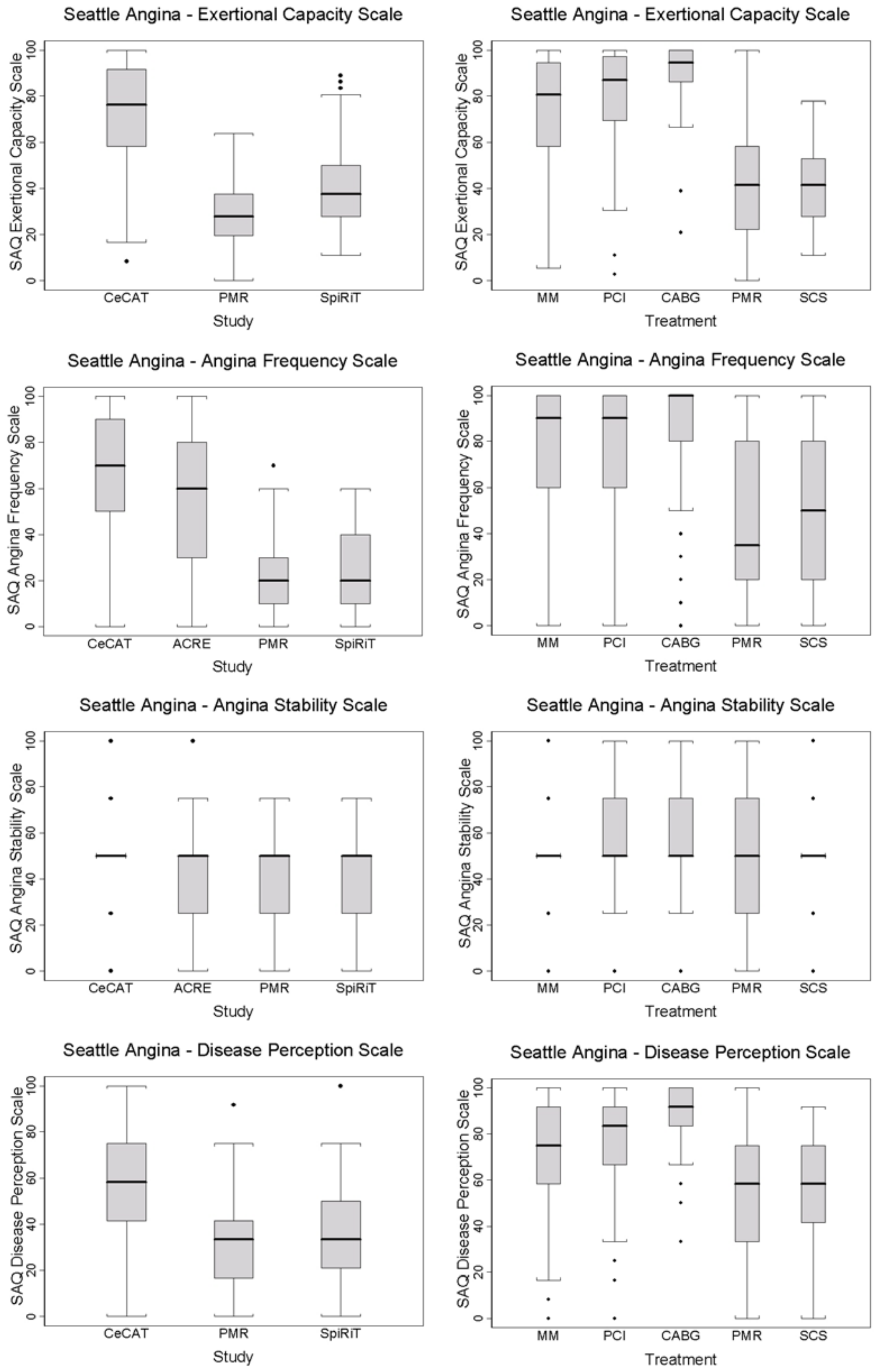

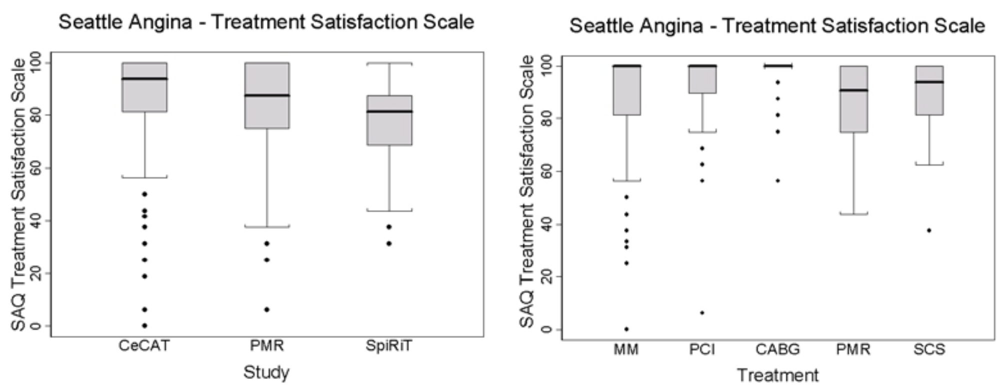

Key: SAQ – Seattle Angina Questionnaire, CeCAT – Cost-effectiveness of functional cardiac testing in the diagnosis and management of coronary heart disease study, PMR – Percutaneous myocardial revascularization compared to continued medical therapy study, SpiRiT – Spinal cord stimulation (SCS) compared to PMR study, ACRE – Appropriateness for coronary revascularization study, MM – medical management, PCI – percutaneous angioplasty/stenting, CABG – coronary artery bypass graft
